# 筛状成分在肺腺癌中的研究进展

**DOI:** 10.3779/j.issn.1009-3419.2020.101.19

**Published:** 2020-07-20

**Authors:** 启峰 丁, 东来 陈, 伟 王, 勇兵 陈

**Affiliations:** 1 215000 苏州，苏州大学附属第二医院胸心外科 Department of Cardiothoracic Surgery, The Second Affiliated Hospital of Soochow University, Suzhou 215000, China; 2 200433 上海，同济大学附属上海市肺科医院胸外科 Department of Thoracic Surgery, Shanghai Pulmonary Hospital, Tongji University School of Medicine, Shanghai 200433, China

**Keywords:** 肺肿瘤, 筛状成分, 预后, Lung neopulasms, Cribriform component, Prognosis

## Abstract

肺癌是全世界发病率最高的恶性肿瘤。目前，肺腺癌（lung adenocarcinoma, LUAD）是肺癌中最常见的组织学类型。然而，具有相同病理亚型的LUAD患者之间却存在显著的预后差异，导致该预后差异的主要原因之一是其组织学异质性。研究表明，除了2015年世界卫生组织（World Health Organization, WHO）对浸润性LUAD的5种主要生长方式的分类外，LUAD中还存在其他的组织学结构并影响其临床预后。其中，筛状成分（cribriform component, CC）是当前LUAD病理组织学的研究热点之一。已有研究表明，CC的有无可进一步预测LUAD患者的预后。随着相关研究逐渐深入，CC与LUAD各组织学类型、临床病理因素、生存预后等方面的关联尚存在争议。本文综合论述了CC在LUAD中的研究进展。

## Abstract (en)

The morbidity of lung cancer ranks top in the world. At present, lung adenocarcinoma (LUAD) is the most common histologic type of lung cancer. However, the prognoses of LUAD patients with the same subtype remain heterogeneous. Histological heterogeneity is one of the main causes of diverse prognoses of patients with LUAD. Studies have shown that there are other histologic patterns that affect the clinical outcomes of LUAD patients, in addition to the five growth patterns of invasive LUAD classified by the World Health Organization (WHO) in 2015. The cribriform component (CC) is one of the research hotspots among histopathology of LUAD. Previous studies have shown that the presence of CC can further stratify the prognoses of patients with LUAD. Along with the progressively deep insights into the aforementioned topic, researchers are dedicating to unveiling the relationships among CC and and other clinicopathological factors as well as their joint influence on the survival of LUAD patients. The review manages to generalize the latest research progress in the CC in LUAD patients.

肺癌是全世界发病率最高的恶性肿瘤^[1]^。目前，肺腺癌（lung adenocarcinoma, LUAD）是肺癌中最常见的组织学类型^[2]^。2011年，国际肺癌研究协会（International Association for the Study of Lung Cancer, IASLC）、美国胸科学会（American Thoracic Society, ATS）和欧洲呼吸学会（European Respiratory Society, ERS）共同提出了一种新的多学科肺癌分类标准^[3]^，将浸润性LUAD按生长方式分为5个主要的病理亚型，即贴壁型、腺泡型、乳头型、微乳头型和实性型，该分类的预后价值和可重复性已在全球范围内得到验证^[4-11]^。因此，2015版世界卫生组织（World Health Organization, WHO）肺肿瘤分类^[12]^中腺癌的分类及诊断标准几乎完全根据2011年IASLC/ATS/ERS提出的LUAD国际多学科分类方案执行。然而，研究发现相同病理亚型的LUAD患者之间的生存预后仍存在显著差异^[13]^，导致该预后差异的主要原因之一是其组织学异质性^[14]^。越来越多的病理学家发现在LUAD病灶中肿瘤细胞的排列形式除常见的5种生长方式外，还可形成其他结构并影响患者的临床预后，如筛状成分（cribriform component, CC）^[15]^和胎儿腺癌成分^[16]^。其中，CC是当前LUAD病理组织学的研究热点之一。近年来，随着对LUAD中CC的不断深入研究，逐步发现了其与临床病理因素的关联性及其对生存预后的影响。本文就CC在LUAD中的研究进展作一综述。

## 1 CC的定义与诊断

Cribriform一词来源于拉丁语Cribrum，意为“筛孔”。Moreira等^[14]^将其定义为具有筛孔状结构的肿瘤细胞巢。CC在涎腺腺样囊性腺癌^[17]^、肺癌^[18]^、乳腺癌^[19]^等多种肿瘤中广泛存在。Kadota等^[20]^进一步将肺癌中的CC明确定义为由于缺乏浸润性生长的肿瘤细胞或间质细胞，导致癌巢中出现无实性成分的筛孔状腺腔（图 1）。与此相反，通常腺泡型LUAD有镜界清楚的单个肿瘤腺体形成的完整腺腔，而实性型LUAD则有实性癌巢且无腺腔形成。目前，CC型腺癌被归为腺泡型腺癌的一个亚型^[21]^，但就组织形态学而言，CC的结构介于腺泡型LUAD和实性型LUAD之间。

**1:**
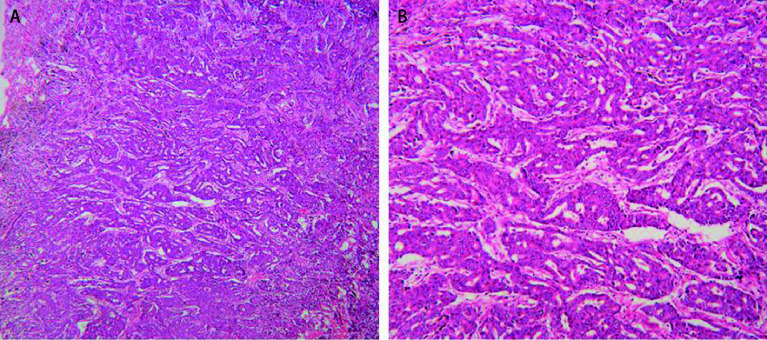
肺腺癌中筛状成分在显微镜下的微观特征。图像均为HE染色。A为放大40倍；B为放大100倍。 Microscopic features of the cribriform component in lung adenocarcinoma. The specimens were stained with hematoxylin and eosin (HE). A: Original ×40; B: Original × 100.

由于对原发性肺肿瘤中的CC认识不足，最初含有这种成分的肺癌通常被认为是转移瘤，特别是来自前列腺癌、乳腺癌和结肠癌的转移瘤^[22-24]^。在1991年，Tsao和Fraser^[25]^首次描述了组织结构与结直肠癌相似的含有CC的原发性肺肿瘤。

CC的诊断主要依赖于组织病理学及免疫组化。在光学显微镜下观察经HE染色的病理组织切片可初步判断标本中是否存在CC，而是否为肺原发性肿瘤则有赖于免疫组化分析。通常认为含有CC的肺癌免疫组化表型为TTF-1（+）/CK7（+）且CK20（-）/CDX-2（-）^[26]^。由于CC的病理形态较为独特，因此能够识别出这种LUAD的组织学成分对于避免误诊为远处转移肿瘤具有重要意义。

## 2 CC在肺腺癌中的组织学分布

在近年的研究^[14, 15, 20, 27-31]^中，含有CC的LUAD患者占入组患者总数的14.9%-39.8%（各项研究病例数如表 1所示）。若将CC作为LUAD的一种组织学亚型，则CC型LUAD患者占入组患者人数的4.2%-11.8%（各项研究病例数如表 1所示），这与微乳头型LUAD患者的出现比例相近^[32, 33]^。一般认为，在LUAD的5种主要组织学亚型中，CC在实性型LUAD中最多见^[14, 15, 20, 27]^，比例可达40.0%（20/50）^[14]^。笔者近期的研究^[31]^发现，CC在含有实性成分的LUAD中比例最高（60.5%, 46/76）。但也有学者认为CC在腺泡型LUAD中比例最高^[29, 30]^，高达67.5%（79/117）^[30]^。而Xu等^[34]^通过回顾性分析125例患者的病理资料认为CC与微乳头成分密切相关，但其他研究^[14, 15, 20, 27-31]^尚不支持CC与微乳头成分的关联。

**1:** 近年来肺腺癌中筛状成分的研究汇总 Summary of the research in lung adenocarcinoma with the cribriform component in recent years

Author	No. of cases	No. of cases with cribriform component	No. of cases with cribriform predominant subtype	Prognostic relevance
Moreira *et al* (2014)^[14]^	249	37 (14.9%)	12 (4.8%)	DFS↓
Kadota *et al* (2014)^[20]^	1, 038	262 (25.2%)	46 (4.4%)	RFP↓
Warth *et al* (2015)^[15]^	674	193 (28.6%)	28 (4.2%)	DFS↓
Kadota *et al* (2018)^[28]^	735	131 (17.8%)	54 (7.3%)	RFP↓、OS↓
Qu *et al* (2018)^[29]^	395	130 (32.9%)	32 (8.1%)	DFS↓
Zhang *et al* (2019)^[30]^	279	111 (39.8%)	33 (11.8%)	OS↓
Ding *et al* (2019)^[31]^	208	67 (32.2%)	-	RFS↓、OS↓
DFS: disease-free survival; RFP: recurrence-free probability; OS: overall survival; RFS: recurrence-free survival.				

## 3 CC相关的临床病理因素

Kadota等^[20]^通过回顾性分析1, 038例患者的临床病理资料首先发现，CC的存在与吸烟史（*P*=0.024）和更高的肿瘤分期（*P* < 0.001）相关。CC的出现也与LUAD侵袭性的病理学表现有关，包括淋巴浸润（*P* < 0.001）、血管浸润（*P* < 0.001）、肿瘤坏死（*P* < 0.001）、更明显的核异型性（*P* < 0.001）、肿瘤细胞增殖旺盛（更多有丝分裂计数）（*P* < 0.001）^[28-30, 34]^。此外，笔者通过对208例LUAD患者的临床病理资料进行回顾性分析发现，含有CC的LUAD更容易发生气腔扩散（spread through air spaces, STAS; *P* < 0.001），STAS可能是导致含有CC的LUAD患者术后预后不良的潜在病理学机制之一^[31]^。其他研究者还发现CC与男性（*P* < 0.001）（*n*=356）^[27]^、肿瘤直径（*P* < 0.001）（*n*=735）^[28]^、淋巴结转移（*P* < 0.001）（*n*=395）^[29]^相关。这些研究结果表明，CC充分展现出浸润性LUAD具有较强侵袭性的生物学特征。

值得注意的是，Warth等^[15]^通过回顾性分析674例患者的临床病理资料后，虽然发现CC在LUAD所有亚型中具有第二高的平均增殖活性（32.6%），介于腺泡型LUAD（23.3%）和实性型LUAD（37.5%）之间，但是其他临床病理变量（性别、年龄、吸烟史、肿瘤分期）均与CC无显著相关。这与之前所述的研究结果并不一致，因此仍然需要更多研究以明确CC与上述临床病理因素的关联。

## 4 CC在分子水平的研究近况

一般来说，表皮生长因子受体（epidermal growth factor receptor, *EGFR*）基因突变在腺泡型LUAD和乳头型LUAD中更常见^[35]^，而*KRAS*突变则在实性型LUAD中更常见^[36]^。Kuang等^[27]^发现CC与*EGFR*基因突变（*P*=0.003）、*AKT1*基因突变（*P*=0.013）和*ALK*基因重排（*P* < 0.001）相关。另一项研究^[15]^表明，在所有组织学亚型中，含有CC的LUAD患者的体细胞*KRAS*基因突变的发生率最高。此外，Kadota等^[28]^还发现表达*ALK*基因的患者比不表达*ALK*基因的患者肿瘤病灶内出现CC的比例更高，尽管其结果没有统计学差异（*P*=0.087）。然而，也有研究^[14, 20, 29, 30]^表明，CC在LUAD患者中与*ALK*基因重排、*EGFR*基因突变和*KRAS*基因突变未见显著相关性。因此，需要更多研究以明确CC与上述基因靶点是否存在相关性。此外，明确含有CC的LUAD中常见突变基因与位点对于术后患者的靶向治疗具有重要意义和研究价值。

## 5 肺腺癌中CC与生存预后的关系

Xu等^[34]^首先提出含有CC的LUAD患者具有较高的淋巴结转移率。Kadota等^[20]^对1, 038例Ⅰ期LUAD患者的临床病理数据进行了回顾性分析，其研究发现：CC型LUAD患者的5年无复发生存率（recurrence-free probability, RFP）为70%，明显低于腺泡型LUAD患者的5年RFP（87%; *P*=0.002）和乳头型LUAD患者的5年RFP（83%; *P*=0.020），但与微乳头型LUAD患者的5年RFP（62%; *P*=0.34）和实性型LUAD患者的5年RFP（70%; *P*=0.56）相仿。肿瘤病灶内CC占比≥10%的LUAD患者的5年RFP显著低于CC占比 < 10%的LUAD患者的5年RFP（73% *vs* 84%; *P* < 0.001）。在多因素分析中，CC占比≥10%的腺泡型LUAD患者的复发风险较CC占比 < 10%的腺泡型LUAD患者的复发风险显著增高（*P*=0.042）。因此，研究者认为CC型LUAD应被视为具有较高复发风险的独特亚型，可区分腺泡型患者中预后较差的亚群。

Kadota等^[28]^在随后的研究中通过回顾性分析735例Ⅰ期-Ⅳ期患者的临床病理资料发现，CC型LUAD患者的5年RFP（51%）均低于贴壁型、腺泡型、乳头型和浸润性黏液型LUAD患者（分别为96%、81%、80%和82%），但与实性型LUAD患者的5年RFP（48%）大致相仿。在多因素分析中，与贴壁型（HR＝0.04, *P* < 0.001）和乳头型及腺泡型（HR＝0.53, P＝0.013）相比，CC型是较差RFP的独立预后因素。在所有患者中，CC型LUAD患者的5年总生存率（overall survival, OS; 49%）均低于贴壁型、腺泡型、乳头型和浸润性黏液型LUAD患者（分别为91%、90%、81%和81%）。在多因素分析中，与贴壁型（HR=0.26, *P* < 0.001）和乳头型及腺泡型（HR=0.46, *P*=0.001）相比CC型是OS的独立风险因素。

此外，Warth等^[15]^通过回顾性分析674例Ⅰ期-Ⅳ期LUAD患者的临床病理资料，证实了CC型LUAD患者相比常见的5种病理亚型的LUAD患者具有更差的无病生存率（disease-free survival, DFS），这与Qu等^[29]^的研究结果一致。在多变量分析中，CC型是LUAD患者DFS不良的独立危险因子（*P*=0.001）。近期的研究^[31]^发现，不仅CC型是患者预后不良的独立预测因素，而且存在CC（肿瘤病灶占比≥5%）亦是较差OS和RFP的独立预测因素。

综上所述，LUAD中存在CC是影响DFS、RFP和OS的重要因素。如表 1所示，所有研究均证实CC对LUAD患者的预后会产生不利影响，但具体影响患者的DFS、RFP还是OS却不尽相同。

## 6 展望

CC作为一种病理组织学成分在LUAD病灶中广泛存在。根据2015年WHO的肺癌分类，CC目前被划分为腺泡型LUAD的一种组织学结构。随着越来越多的研究证实CC可进一步分层LUAD患者的预后，CC能否独立成为LUAD的一种组织学亚型以及能否对下一版LUAD病理分类产生影响值得期待。尽管我们近期的研究发现含有CC的LUAD更容易发生STAS，但CC是否能通过其他途径，如淋巴结微转移（lymph node micrometastasis, LNMM）或脏层胸膜浸润（visceral pleural infiltration, VPI）影响LUAD患者的生存预后以及具体与哪项预后指标（OS、DFS和/或RFP）相关联均尚无定论，还需要多中心大样本量的临床数据进行荟萃分析得以论证。

目前的临床研究证据证明，CC的临床重要性尚未得到充分重视。由于通过观察经HE染色的病理组织切片即可初步诊断是否存在CC，因此，病理科医师应该尝试探索在冰冻切片中识别CC并在术中快速病理报告中报告该成分，以便指导外科术式。对于已行肺楔形切除且术中快速病理提示含有CC的患者，是否需要继续行解剖性肺段切除或肺叶切除以及肺段切除与肺叶切除的生存预后是否存在差异仍有待讨论。对于含有CC的Ⅰ期LUAD患者术后是否需要进行辅助化疗亦不得而知。同时，辅助放射治疗能否使含有CC的Ⅲa-N2期患者产生生存获益，这是将来值得探究的方向。此外，研究发现CC与*EGFR*基因突变、*AKT1*基因突变、*KRAS*基因突变和*ALK*基因重排相关，但与上述突变基因相关的下游通路活化情况及分子机制尚不明确，可通过基因芯片或高通量测序等技术为含有CC的LUAD患者发掘出潜在治疗靶点。

综上所述，需要更多前瞻性研究和分子生物学实验，以期为含有CC的LUAD患者的手术方式和治疗模式的选择提供理论依据，使患者最大获益。
